# Delivery of Doxorubicin by Ferric Ion-Modified Mesoporous Polydopamine Nanoparticles and Anticancer Activity against HCT-116 Cells In Vitro

**DOI:** 10.3390/ijms24076854

**Published:** 2023-04-06

**Authors:** Mengwen Guo, Junhong Ling, Xinyi Xu, Xiaokun Ouyang

**Affiliations:** School of Food and Pharmacy, Zhejiang Ocean University, Zhoushan 316022, China

**Keywords:** mesoporous polydopamine, ferric ions, doxorubicin (DOX), hyaluronic acid target modification

## Abstract

In clinical cancer research, photothermal therapy is one of the most effective ways to increase sensitivity to chemotherapy. Here, we present a simple and effective method for developing a nanotherapeutic agent for chemotherapy combined with photothermal therapy. The nanotherapeutic agent mesoporous polydopamine-Fe(III)-doxorubicin-hyaluronic acid (MPDA-Fe(III)-DOX-HA) was composed of mesoporous polydopamine modified by ferric ions and loaded with the anticancer drug doxorubicin (DOX), as well as an outer layer coating of hyaluronic acid. The pore size of the mesoporous polydopamine was larger than that of the common polydopamine nanoparticles, and the particle size of MPDA-Fe(III)-DOX-HA nanoparticles was 179 ± 19 nm. With the presence of ferric ions, the heat generation effect of the MPDA-Fe(III)-DOX-HA nanoparticles in the near-infrared light at 808 nm was enhanced. In addition, the experimental findings revealed that the active targeting of hyaluronic acid to tumor cells mitigated the toxicity of DOX on normal cells. Furthermore, under 808 nm illumination, the MPDA-Fe(III)-DOX-HA nanoparticles demonstrated potent cytotoxicity to HCT-116 cells, indicating a good anti-tumor effect in vitro. Therefore, the system developed in this work merits further investigation as a potential nanotherapeutic platform for photothermal treatment of cancer.

## 1. Introduction

Colon cancer is a disease with a high morbidity and mortality rate worldwide, which may be attributable to poor diet and a reversed work schedule [1,2]. Although academic research on colon cancer treatment has never stopped, the incidence of colon cancer continues to rise [3,4,5]. Currently, the primary anticancer treatments include surgery, chemotherapy, and radiotherapy [6,7,8]. Chemotherapy using doxorubicin (DOX), a broad-spectrum anthracycline antitumor drug, is widely used to develop model drugs for tumor-targeted drug delivery systems [9,10,11,12]. However, a single chemotherapy regimen can lead to several serious side effects, cancer metastasis, and tumor resistance [13,14]. Studies have demonstrated that chemotherapy combined with photothermal therapy (PTT) can reduce the heat resistance of tumor cells and that the heat generated by PTT can alleviate tumor hypoxia to further promote chemotherapy [15,16,17]. Thus, combined photothermal and chemotherapeutic treatment can effectively mitigate the drawbacks of monotherapy. With the ongoing development of nanotechnology, it has been found that nanodrug delivery systems can be used for targeted drug delivery to the tumor site, reduce dosage, and enhance the anticancer activity of chemotherapeutic drugs [18,19,20]. This type of loading system is typically biocompatible, degradable, and modifiable [21,22].

Polydopamine (PDA), a natural melanin polymer formed by the self-aggregation of dopamine (DA) [23], can be decomposed in the weakly acidic tumor microenvironment [24]. PDA has many applications in multifunctional surface modification due to its natural nontoxicity, biodegradability, and high absorptivity in the near-infrared region (NIR) [25]. Therefore, PDA has numerous applications in tumor photothermal therapy. However, the lack of photothermal performance hinders its chemotherapeutic effects. Studies have shown that PDA strongly chelates metal ions, and the addition of metal ions to PDA can significantly improve the photothermal conversion efficiency of PDA nanoparticles [26].

In addition, we selected mesoporous polydopamine nanoparticles (MPDA) as drug carriers to increase the loading rate of chemotherapeutic drugs. With a specific surface area and pore size, MPDA nanoparticles can effectively increase the loading capacity of chemotherapeutic drugs [27]. During drug administration, mesoporous nanoparticles exhibit severe drug leakage. Therefore, an appropriate encapsulant is typically used when designing the drug delivery system [28]. Hyaluronic acid (HA) is highly hydrophilic, which can increase the stability of MPDA nanoparticles and also has a targeting effect due to the expression of specific receptors on the surface of tumor cells [29]. As an encapsulant, HA can therefore reduce drug loss and cytotoxicity.

**Figure 1:**
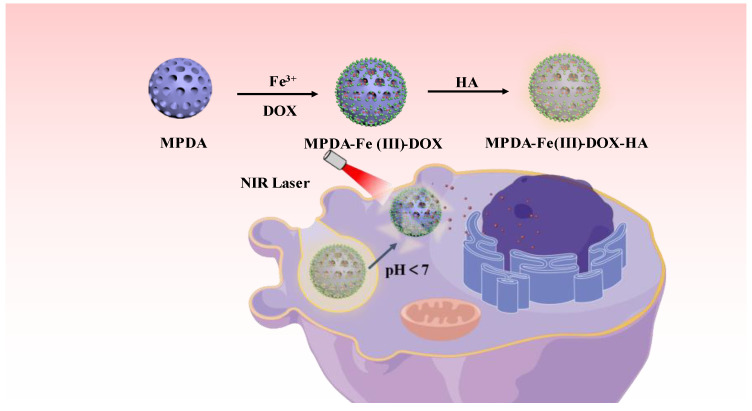
Schematic representation of the preparation of MPDA-Fe(III)-DOX-HA nanoparticles and their anti-tumor activity.

Here, we designed and synthesized a photothermal–chemotherapy combined drug delivery system (MPDA-Fe(III)-DOX-HA) by introducing ferric ions to enhance the photothermal effect of the mesoporous PDA nanoparticles loaded with the chemotherapeutic drug doxorubicin and encapsulated with hyaluronic acid. The simplify preparation process is shown in Figure 1.

MPDA-Fe(III)-DOX-HA nanoparticles were gathered near tumor cells via the active target of HA. Due to the sensitivity of tumor cells to temperature, 808 nm near-infrared light irradiation caused polydopamine nanoparticles to generate a substantial amount of heat and inhibit the growth of tumors. Subsequently, PDA disintegrated in a weakly acidic environment, releasing the chemotherapeutic drug doxorubicin, which, when combined with the photothermal effect, killed tumor cells. In summary, the MPDA-Fe(III)-DOX-HA delivery system not only increased the efficacy of chemotherapy but also decreased cytotoxicity, indicating that photothermal combined with chemotherapy is a promising strategy for treating tumors and that tumors can be destroyed by the synergistic effect of the two treatments.

## 2. Results

### 2.1. BET Analysis

**Figure 2:**
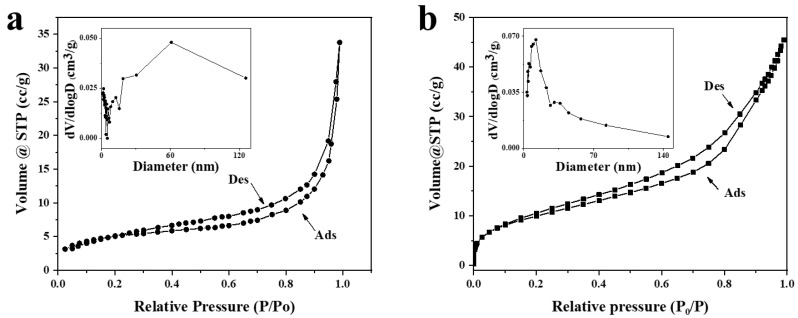
N_2_ Adsorption and desorption isotherms, as well as size analysis of PDA nanoparticles (**a**) and MPDA nanoparticles (**b**). The abbreviations Ads and Des in the figures refer to adsorption and desorption, respectively.

The N_2_ adsorption–desorption isotherms of PDA and MPDA were measured (Figure 2a,b, with the pore size distribution in the top right corner). Both PDA and MPDA had typical Langmuir IV isotherms, as shown in Figure 2a,b, indicating that both PDA and MPDA may have pore structures [30]. The pore size curve of PDA in Figure 2a shows that the existing pore structure may have larger pores caused by mutual adhesion and polymerization of PDA, so the pore content of mesopores is relatively low. In Figure 2b, the specific surface area of MPDA is shown to be 36.824 m^2^g^−1^, which is significantly larger than that of non-mesoporous PDA spheres (17.126 m^2^g^−1^), indicating that MPDA is more suitable for drug loading as a drug carrier than PDA.

As shown in Figure 2, an H4-type hysteresis loop caused by capillary agglomeration occurs in the P/P_0_ range of 0.2–0.9. The average pore size of MPDA is 3.827 nm (Figure 2b), and that of PDA is 1.347 nm (Figure 2a), with poor pore size distribution. Based on these findings, it can be concluded that the mesoporous structure of MPDA can provide a larger specific surface area for drug loading and improve the drug loading capacity.

### 2.2. SEM and Particle Size Analysis

**Figure 3:**
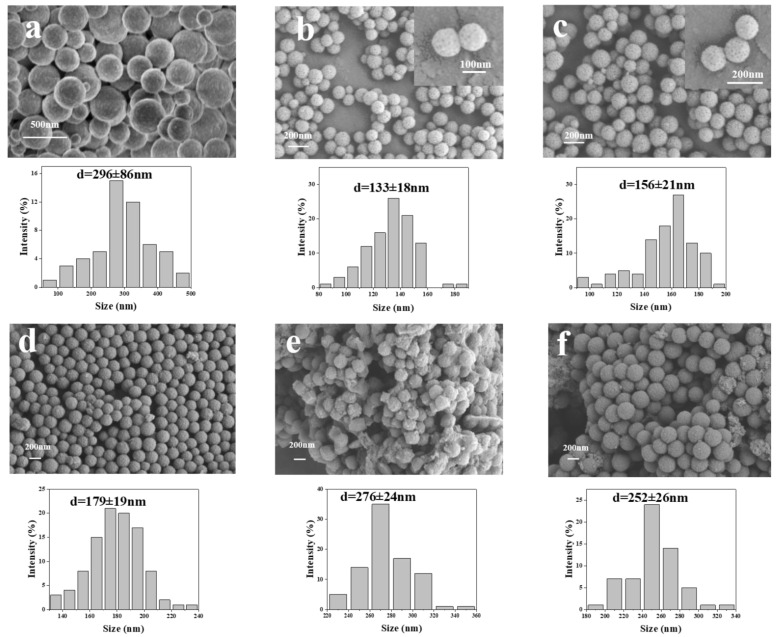
SEM and particle size analysis template-free dopamine nanoparticles (**a**), acetone–ethanol elution dopamine nanoparticles (**b**), anhydrous ethanol elution dopamine nanoparticles (**c**), m (MPDA-Fe(III)-DOX:HA) = 1:1 (**d**), m (MPDA-Fe(III)-DOX:HA) = 1:2 (**e**), m (MPDA-Fe(III)-DOX:HA) = 1:3 (**f**)).

SEM images of various nanomaterials obtained in the experiment are shown in Figure 3. Results indicate that the addition of TMB could optimize the preparation of MPDA. Figure 3a shows that the PDA nanoparticles without the TMB template lack a mesoporous structure and have a non-uniform particle size distribution with an average particle size of 296 nm. The MPDA particles prepared with TMB have a distinct mesoporous structure and a uniform particle size. The results of optimizing the elution conditions of the template are shown in Figure 3b,c. MPDA nanoparticles with a relatively uniform distribution and small particle size (133 ± 18 nm) were obtained in the studies using acetone–ethanol and ethanol as the elution templates and acetone–ethanol as the eluent (Figure 3b). In contrast, the MPDA nanoparticles (Figure 3c) prepared after the removal of the template using ethanol as the eluent had a non-uniform particle size distribution and a mean particle size of 156 ± 21 nm. Therefore, the acetone–ethanol elution condition was selected as the subsequent elution condition.

In this study, the effect of HA modification at different proportions on the particle size of MPDA drug-loaded nanoparticles was investigated. When comparing Figure 3d–f to Figure 3b, HA was successfully coated on the MPDA particles. The HA-modified MPDA nanoparticles are distributed evenly because the hydrophilicity of HA improves the dispersibility of nanoparticles in the solution. Figure 3d–f correspond to m (MPDA-Fe(III)-DOX:HA = 1:1), m (MPDA-Fe(III)-DOX:HA = 1:2), and m (MPDA-Fe(III)-DOX:HA = 1:3), respectively. When m (MPDA-Fe(III)-DOX:HA = 1:1) was used instead of m (MPDA-Fe(III)-DOX:HA = 1:2, 1:3), the prepared nanoparticles were more evenly distributed with an average particle size of 179 ± 19 nm. Therefore, m (MPDA-Fe(III)-DOX:HA = 1:1) was selected for the preparation of the MPDA nanoparticles as drug carriers.

### 2.3. Zeta Potential Analysis

**Figure 4:**
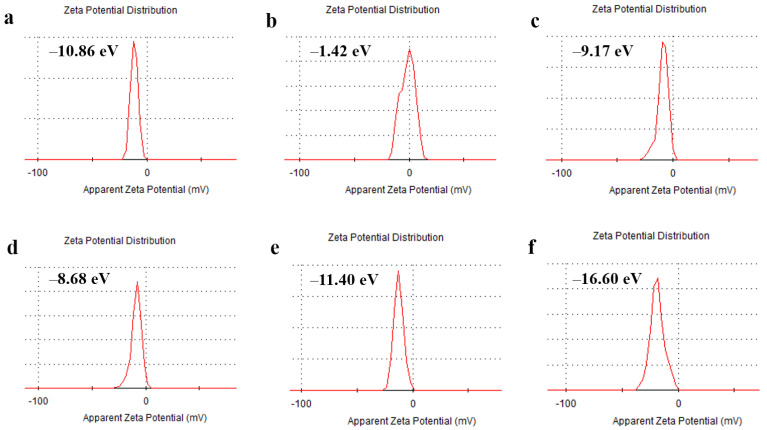
Zeta potentials of (**a**) MPDA, (**b**) MPDA-Fe(III)-DOX, (**c**) MPDA-Fe(III)-DOX-HA, (**d**) m (MPDA-Fe(III)-DOX): m (HA) = 1:1, (**e**) m (MPDA-Fe(III)-DOX): m (HA) = 1:2, (**f**) m (MPDA-Fe(III)-DOX): m (HA) = 1:3.

As shown in Figure 4a, the surface potential of MPDA was −10.86 mV. After doxorubicin was loaded into MPDA, the surface potential of MPDA-Fe(III)-DOX increased to −1.42 mV (Figure 4b), which may be because the negative charge of the MPDA carrier itself was significantly reduced due to the chelation of metal ions on the surface and the loading of DOX drugs. The surface electronegativity of MPDA nanoparticles was significantly increased after HA modification due to the strong electronegativity of the carboxyl group in sodium hyaluronate. As shown in Figure 4b, the surface potential of MPDA-Fe(III)-DOX-HA modified by HA was more electronegative than that of MPDA-Fe(III)-DOX, reaching −9.17 mV (Figure 4b). The zeta potential of MPDA modified with HA was also investigated. As shown in Figure 4d–f, the zeta potential of the HA-modified MPDA nanoparticles was deprotonated by the carboxyl group on the HA surface. The electronegativity of MPDA nanoparticles with a higher proportion of HA modification was higher, indicating that HA was successfully modified on the surface of MPDA.

### 2.4. FTIR Analysis

**Figure 5:**
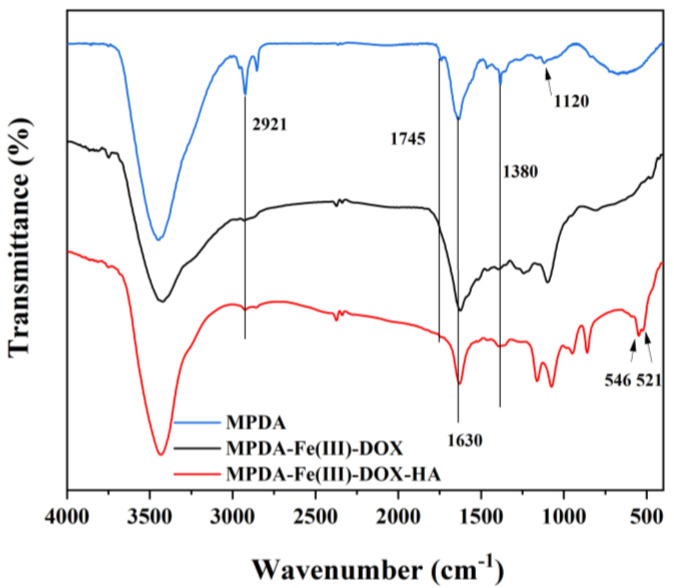
Comparison of the IR spectra of nanoparticles at different steps.

The infrared absorption of MPDA, MPDA-Fe(III)-DOX, and MPDA-Fe(III)-DOX-HA nanoparticles was investigated using infrared spectroscopy. As shown in Figure 5, the absorption peaks of PDA are at −1630 cm^−1^ (the telescopic vibration peak of the aromatic ring and the bending vibration peak of N–H) [31], −1380 cm^−1^ (the phenolic C–O–H bending vibration), −1120 cm^−1^(C–O vibration) [32], and 2921 cm^−1^ (the C–H telescopic vibration peak caused by aromatic and aliphatic C–H) [33]. This further indicates that PDA is prepared. The absorption peak at 1745 cm^−1^ could be attributed to the C=O stretching vibration peak [34]. The peak intensities at 2921 cm^−1^ and 1745 cm^−1^ were significantly reduced after DOX loading and HA modification, which was due to the reduction of the aldehyde group caused by the participation of Fe in chelation after DOX loading. The bands at 546 and 521 cm^−1^ in MPDA-Fe(III)-DOX-HA are attributed to the elastic and contractile vibration peaks of Fe–O [35], indicating that some free Fe ions may be involved in the chelation of HA and that the HA layer on the drug-loaded nanoparticles has been modified.

### 2.5. XPS Analysis

**Figure 6:**
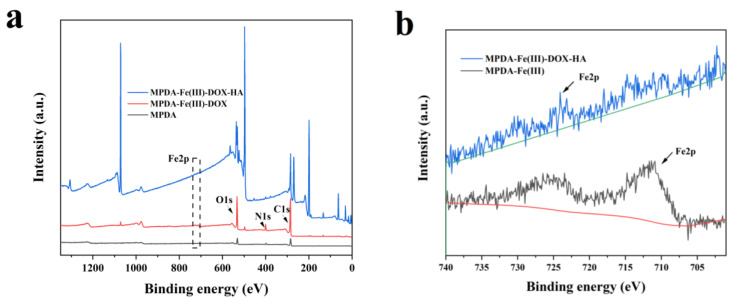
Spectrum of XPS (**a**), spectral comparison of Fe 2p in XPS before and after HA inclusion (**b**). The green and red lines in (**b**) are both background lines.

The X-ray photoelectron spectroscopy results of MPDA, MPDA-Fe(III)-DOX, and MPDA-Fe(III)-DOX-HA are shown in Figure 6a,b. The full spectrum in Figure 6a shows that each material contains C, N, and O elements [36]. Because of the relatively low content of Fe, we further analyzed the Fe 2p spectra of MPDA-Fe(III)-DOX-HA and MPDA-Fe(III). The peak in MPDA-Fe(III) indicates that Fe was chelated successfully on MPDA [37]. The Fe content of MPDA-Fe(III)-DOX-HA decreased, which could be attributed to the relatively low Fe content on the surface of HA-modified nanoparticles.

### 2.6. Photothermal Conversion Capability Analysis

**Figure 7:**
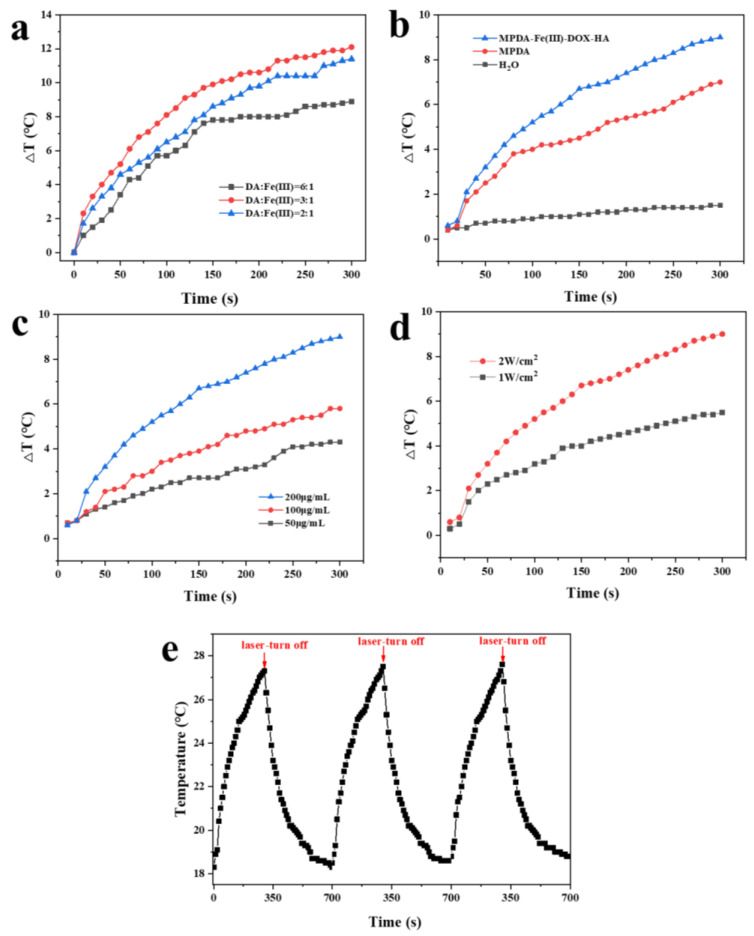
After 5 min of 2 W/cm^2^ NIR laser irradiation, the temperature difference curve of different DA:Fe molar ratio (**a**), materials (**b**), and MPDA-Fe(III)-DOX-HA concentration (**c**). Temperature difference curve for a 1 mg/mL MPDA-Fe(III)-DOX-HA solution at various laser powers (**d**). The temperature change per 10 s of 5 min irradiation by a 2 W/cm^2^ NIR laser after three cycles of cooling to room temperature (**e**).

As a photothermal agent, PDA has a strong near-infrared absorption capacity and an absorption capacity in the 808 nm near-infrared band [38]. Ferric ions were added to the MPDA preparation process to improve the infrared absorption capacity and photothermal efficiency of the obtained MPDA nanoparticles [39]. In this study, the ferric ion addition ratio was optimized. The effects of different ferric ion addition ratios on the photothermal efficiency of MPDA nanoparticles were investigated under irradiation conditions of 808 nm and 2 W/cm^2^. Figure 7a shows that the prepared nanoparticles had the best heating effect when the dopamine (DA):Fe ratio was 3:1.

Figure 7b shows the photothermal experiments on samples from various preparation steps. MPDA-Fe(III)-DOX-HA demonstrated good photothermal conversion ability, whereas the pure water temperature in the control group changed slightly. Under the irradiation of different powers of near-infrared laser (Figure 7c) and 808 nm near-infrared light for 10 min (1 W/cm^2^ and 2 W/cm^2^), the 2 W/cm^2^ laser exhibited a superior photothermal conversion effect.

The photothermal performance of MPDA-Fe(III)-DOX-HA was further evaluated by dispersing MPDA-Fe(III)-DOX-HA in an aqueous solution at varying concentrations (50, 100, and 200 μg/mL). As shown in Figure 7c, the temperature of the MPDA-Fe(III)-DOX-HA solution varied in a concentration-dependent manner. The increase in temperature of the MPDA-Fe(III)-DOX-HA solution (200 μg/mL) from 18.3 °C to 27.3 °C indicates that MPDA-Fe(III)-DOX-HA could effectively convert near-infrared light into thermal energy.

In this study, the photothermal stability of MPDA-Fe(III)-DOX-HA was further evaluated using cyclic laser irradiation. As shown in Figure 7e, the highest temperature reached by MPDA-Fe(III)-DOX-HA was relatively stable after three cycles of laser irradiation, indicating that MPDA-Fe(III)-DOX-HA possessed good photothermal stability.

### 2.7. DOX Loading and Release Analysis

Table 1 shows that when the DA to Fe(III) molar ratio is 6:1, 3:1, or 2:1, the drug loading rate and encapsulation efficiency of the obtained nano-sized drug-loaded particles (MPDA-Fe(III)-DOX-HA) are 80.41 ± 0.84%, 16.08 ± 0.16%; 84.90 ± 0.68%, 16.98 ± 0.13%; and 81.87 ± 1.26%, 16.35 ± 0.25%, respectively. The loading effect of MPDA-Fe(III)-DOX-HA was optimal when the DA to Fe(III) molar ratio was 3:1.

**Figure 8:**
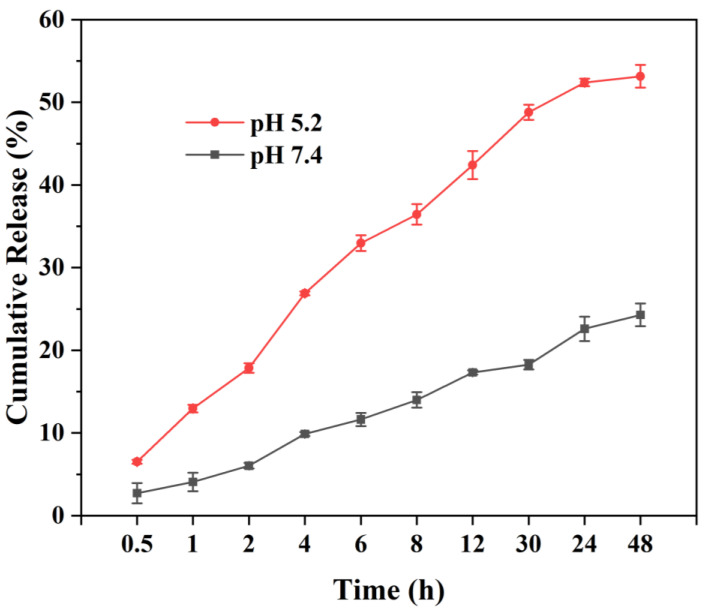
The ability of MPDA-Fe(III)-DOX-HA to release DOX in different pH environments.

To investigate the drug release behavior of MPDA-Fe(III)-DOX-HA, PBS and ABS buffer were used to simulate the internal body environment and the tumor microenvironment, respectively. As shown in Figure 8, the drug release rate increased by nearly 30% to 53.1% under the simulated tumor microenvironment when compared to the normal PBS environment. This indicates that MPDA-Fe(III)-DOX-HA had a more potent disintegration and release capacity in acidic environments, which may be due to the instability of the dopamine structure in an acidic solution, which increased the drug release [40]. Additionally, protonation of amine groups in DOX at acidic pH results in higher solubility of DOX and faster drug release [41].

### 2.8. Cytotoxicity Analysis

**Figure 9:**
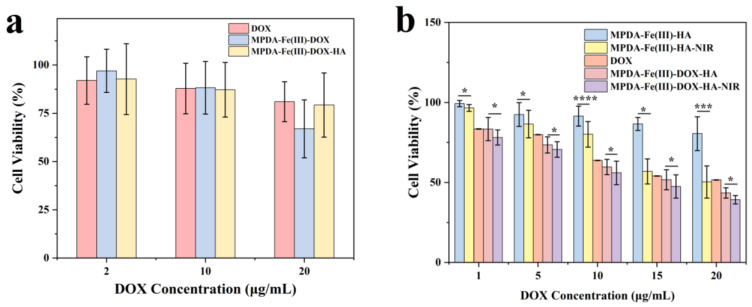
Cytotoxicity analysis using the MTT method (toxicity of different concentrations of drug delivery system on L929 cells (**a**), toxicity of different concentrations of drug delivery system to HCT-116 cells under different NIR (**b**)) (* *p* < 0.05; *** *p* < 0.001; **** *p* < 0.0001).

Nanodrug carriers are used for drug delivery, and their toxicity should be determined [42]. In this study, the MTT assay was used to determine the toxicity of DOX, MPDA-Fe(III)-DOX, and MPDA-Fe(III)-DOX-HA on L929 and HCT-116 cells. As shown in Figure 9a, freely available DOX, MPDA-Fe(III)-DOX, and MPDA-Fe(III)-DOX-HA had no significant toxicity to mouse fibroblasts. The survival rate of cells receiving MPDA-Fe(III)-DOX was 66.94% at a DOX concentration of 20 μg/mL, which may be attributed to the targeting effect of MPDA-Fe(III)-DOX without a modification layer in a normal cell culture environment [43]. DOX release was uncontrolled, so its release capacity was high, resulting in increased toxicity to normal cells [44]. The survival rate of MPDA-Fe(III)-DOX increased to 79.28% after HA modification. Survival rates were greater than 80% at the remaining DOX concentrations.

The toxicity of varying DOX concentrations (calculated by release rate) was evaluated using HCT-116 cells. The results indicated that the drug-loaded nanoparticles MPDA-Fe(III)-HA had no significant toxicity on tumor cells (survival rate > 80%), as shown in Figure 9b, but the survival rate in the corresponding MPDA-Fe(III)-HA-NIR group was only 50.29% at a DOX concentration of 20 μg/mL, due to the photothermal properties of the nanoparticles. As demonstrated in the MPDA-Fe(III)-DOX-HA experimental group, at a DOX concentration of 20 μg/mL, the tumor cell viability was significantly different in the NIR group, indicating that MPDA-Fe(III)-DOX-HA had good photothermal properties and could work in conjunction with DOX to kill tumor cells (Figure 9b). At this time, the tumor cell viability was reduced to 39.1%. In comparison to the free DOX group and the MPDA-Fe(III)-DOX-HA group, the MPDA-Fe(III)-DOX-HA-NIR group exhibited significant inhibition at the same drug concentration. The enhanced cytotoxicity was caused by the thermal effect generated by near-infrared radiation in combination with the action of DOX.

### 2.9. Cellular Uptake Analysis

**Figure 10:**
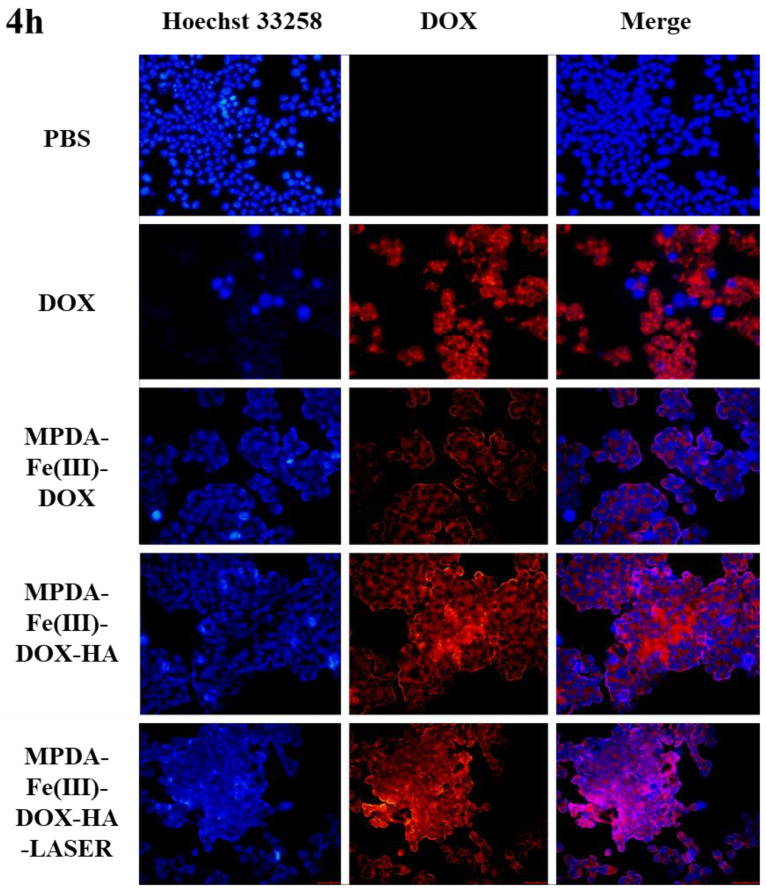

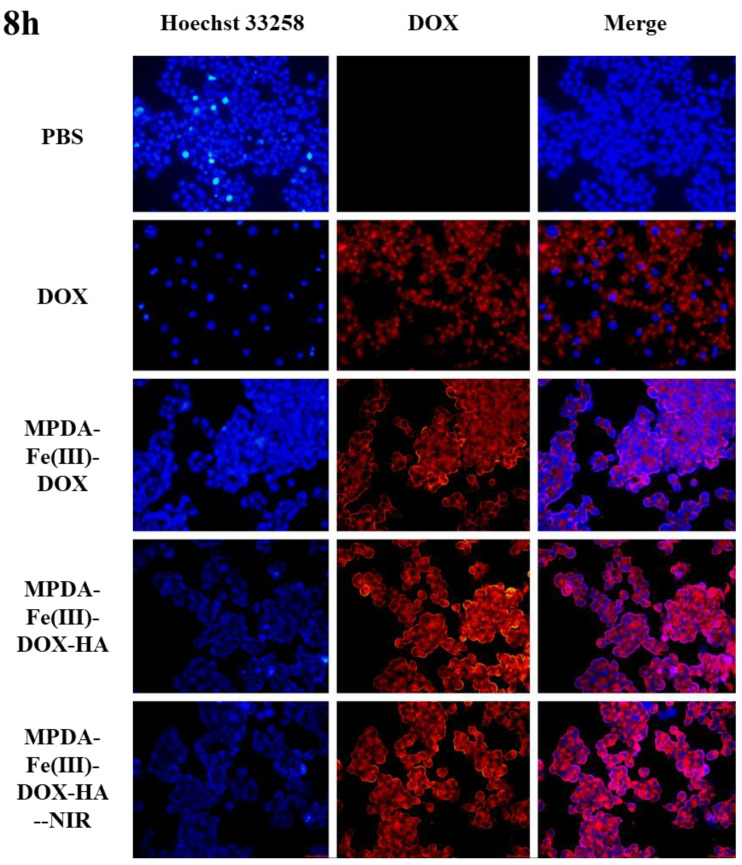
The cell survival and uptake capacity of the free DOX, MPDA-Fe(III)-DOX, MPDA-Fe(III)-DOX-HA, and MPDA-Fe(III)-DOX-HA-NIR groups cultured in HCT-116 cells for 4 h and 8 h at the same DOX concentration.

This study investigated the distribution of DOX after 4 h and 8 h of incubation of HCT-116 cells with drug-loaded nanoparticles, as well as the tumor cell uptake of MPDA-Fe(III)-DOX-HA nanoparticles. CD44 receptors are highly expressed in HCT-116 cells [45]. Tumor cell uptake of MPDA-Fe(III)-DOX (without HA modification) and MPDA-Fe(III)-DOX-HA (HA-modified) nanoparticles were compared. In addition, the differences in nanoparticle uptake behavior with and without near-infrared light irradiation were investigated. As shown in Figure 10, the fluorescence intensity of HCT-116 cells treated with Hoechst 33,258 increased with culture time. At the same time, the fluorescence intensity of DOX in cells increased over time, indicating that the nanoparticles were ingested rather than attached to the cell surface. In addition, when compared to MPDA-Fe(III)-DOX nanoparticles, MPDA-Fe(III)-DOX-HA demonstrated higher DOX fluorescence intensity at varying uptake times, indicating that the HA-modified nanoparticles could enhance nanoparticle uptake by tumor cells. The DOX fluorescence intensity of tumor cells in the near-infrared light irradiation group was higher than in the group without NIR light irradiation because the heat generated after the near-infrared light irradiation could promote drug release by the nanocarriers [46].

## 3. Discussion

Overall, our studies establish that the MPDA-Fe(III) prepared by adding trivalent ferric ions significantly improved the photothermal effect of MPDA, which was consistent with the conclusion in the relevant literature that metal ions enhanced the photothermal effect of polymers [47]. However, we found that the photo-conversion of MPDA-Fe(III)-DOX-HA was lower than that of MPDA-Fe(III), possibly due to the chelation of some iron ions by HA. However, photothermal cycling experiments showed that MPDA-Fe(III)-DOX-HA still had good photothermal stability.

In addition, we have found in the cell experiment that the mesoporous polydopamine has good biocompatibility, and the killing effects of free doxorubicin within a certain concentration range on normal cells and cancer cells are different, which may be related to the action mechanism of doxorubicin. At the same time, the cell experiment also showed the targeting effect of hyaluronic acid. The material encapsulated by hyaluronic acid could reduce the toxic effect on L929 cells. In addition, experiments on cancer cell HCT-116 cells proved that MPDA-Fe(III)-DOX-HA had a good killing effect on tumor cells, and the differential expression of this nanodrug delivery system in normal cells indicated that it is a potential good platform for cancer drug delivery.

## 4. Materials and Methods

### 4.1. Materials

Doxorubicin hydrochloride (DOX · HCl, 98%) was purchased from Shanghai Haoyun Chem Technology Co., Ltd. (Shanghai, China) F127 (Biochemical Reagent, BR) was purchased from Sigma-Aldrich (Shanghai, China) Trading Co., Ltd. (Shunan, Japan) Hyaluronic acid (97%, 40 KDa–100 KDa) was purchased from Shanghai Macklin Biochemical Co., Ltd. (Shanghai, China) Ammonia (NH_3_·H_2_O, Analytical Reagent, AR) was purchased from Sinopharm Chemical Reagent Co., Ltd. (Shanghai, China) Dopamine hydrochloride (DA · HCl, 98%), 1,3,5-trimethylbencene (TMB, AR, 97%), and FeCl_3_·6H_2_O (AR, 99%) were purchased from Shanghai Aladdin Bio-Chem Technology Co., Ltd. (Shanghai, China) L929 mouse epithelial cells (SCSP-5039) and HCT-116 cells (TCHu 99) were purchased from National Collection of Authenticated Cell Cultures.

### 4.2. Synthesis of Mesoporous Polydopamine (MPDA) Nanoparticles

Mesoporous polydopamine (MPDA) nanoparticles were prepared using the one-pot method [48], and in the classic experiment, TMB was added to optimize the preparation of MPDA nanoparticles. First, 250 mg of F127 and 100 μL of TMB were added to 10 mL of 50% ethanol. After 5 min of ultrasonic treatment, 75 mg of DA · HCl was added, followed by 450 μL of ammonia to adjust the pH value. The mixture was magnetically stirred for 24 h before being centrifuged at 12,000 rpm for 12 min at 25 °C. The precipitate was washed three times with acetone:ethanol (1:3; *v*:*v*). To compare the effect of TMB on MPDA nanoparticles, MPDA nanoparticles without TMB were prepared without changing any other parameters.

### 4.3. Preparation of MPDA-Fe(III)-DOX Nanoparticles

The MPDA nanoparticles were first prepared according to the molar ratios (DA:Fe) of 6:1, 3:1, and 2:1. The obtained MPDA nanoparticles and ferric chloride were dissolved in the above molar ratio in PBS and vortexed at 1000 rpm for 5 min to obtain three different iron-crosslinked MPDA (MPDA-Fe(III)) nanoparticles. DOX in various concentrations was dissolved in PBS, and the three ferric crosslinked MPDA nanoparticles were added in turn. The supernatant was obtained by centrifugation at 11,000 rpm for 10 min. The performance of the three ferric crosslinked loaded DOX was evaluated, and the best one, MPDA-Fe(III) nanoparticle-loaded DOX, was optimized to obtain MPDA-Fe(III)-DOX for the subsequent experiments.

### 4.4. Hyaluronic Acid-Based Modification of Nanoparticles

In the experiment, the optimal MPDA-Fe(III)-DOX was used for HA modification, and the mass ratios of MPDA-Fe(III)-DOX to HA were 1:1, 1:2, and 1:3. High-speed centrifugation was used after thorough mixing to obtain MPDA-Fe(III)-DOX-HA modified with varying proportions of HA. Zeta potential values and SEM images were evaluated to obtain the optimal proportion of HA.

### 4.5. Characterization of Nanocarrier

#### 4.5.1. Brunner–Emmett–Teller (BET) Measurements

In this study, the IQ3 automatic specific surface and porosity analyzer was used to determine the specific surface area and pore volume of the nanocarrier via adsorption. The N_2_ adsorption–desorption isotherms were determined in continuous adsorption mode at 77.35 K, and the specific surface area, pore size, and pore volume were determined using BET and BJH methods.

#### 4.5.2. Scanning Electron Microscopy (SEM)

The nanoparticles were dispersed in an ethanol solution, sampled, dropped onto tin-foil paper, and sprayed with gold after natural drying. The morphology of the nanoparticles was examined under a scanning electron microscope.

#### 4.5.3. Fourier Transform Infrared Spectroscopy (FTIR)

Experimentally obtained nanoparticles were dried and sample-prepared before infrared spectrum analysis using the Bruker Tensor II infrared spectrometer.

#### 4.5.4. X-ray Photoelectron Spectroscopy (XPS)

The X-ray photoelectron spectrum of the nanocarrier was analyzed, and the changes in the energy spectrum of the nanocarrier were compared before and after drug loading.

#### 4.5.5. DOX Loading and In Vitro Release

1. Standard curve

The standard curve of DOX was plotted using ultraviolet spectrophotometry. Following the preparation of 10 mg DOX in 1 mg/mL mother solution with H_2_O, the mother solutions were diluted to obtain 2, 4, 8, 10, and 16 μg/mL DOX solutions. At 480 nm, the absorbance value was measured, and the standard curve of DOX was plotted.

2. Loading rate and encapsulation efficiency

The nanocarrier was added to a 100 μg/mL DOX solution, oscillated and loaded overnight, and centrifuged at 11,000 rpm for 10 min. The supernatant was taken to determine DOX content. The centrifuged precipitate was lyophilized and weighed. The loading rate and encapsulation efficiency of MPDA-Fe(III) nanoparticles were calculated.

The loading capacity (LC) and encapsulation efficiency (EE) were calculated using the following formulas:

(1) $$\mathrm{LC}=\frac{W_{0}-W_{1}}{W_{2}}\times 100\%$$

(2) $$\mathrm{EE}=\frac{W_{0}-W_{1}}{W_{0}}\times 100\%$$

The *W*_0_, *W*_1_, and *W*_2_ represent the initial DOX addition, the DOX content of the supernatant after centrifugation, and the weight of the centrifuged precipitate after freeze-drying, respectively.

#### 4.5.6. In Vitro Release

The release capacity of MPDA-Fe(III)-DOX-HA at different pH values was determined using dialysis. The dialysis bag (MW = 3500) was packed with the same amount of MPDA-Fe(III)-DOX-HA in PBS (pH = 7.4) or acetic acid buffer (pH = 5.2). After sealing, it was dispersed in the corresponding 200 mL buffer to simulate the normal human internal environment and tumor microenvironment, and the release process of the drugs in vivo was examined. Under constant temperature oscillation at 150 rpm and 37 °C, 2 mL of dialysate in the beaker was collected, and 2 mL of the corresponding buffer was added at a certain time point for continuous release for 48 h. The DOX content of the dialysate was measured, and the ratio to the initial DOX content was calculated to plot the release curve.

#### 4.5.7. Photothermal Conversion Efficiency

MPDA, MPDA-Fe(III)-DOX, and MPDA-Fe(III)-DOX-HA solutions were prepared in 100 μL deionized water at a concentration of 200 μg/mL, and the temperature rise was measured under near-infrared laser irradiation (2 W/cm^2^, 5 min). The nanomaterial solutions with different concentrations (50, 100, and 200 μg/mL) and power densities (1 and 2 W/cm^2^) were irradiated for 5 min to evaluate the photothermal effects at different irradiation powers. The 200 μg/mL MPDA-Fe(III)-DOX-HA solution was irradiated with a near-infrared laser (2 W/cm^2^) for 5 min and allowed to naturally cool to 25 °C. The photothermal conversion rate of MPDA-Fe(III)-DOX-HA was then calculated.

#### 4.5.8. Cytotoxicity

The MTT assay was used to evaluate the cytotoxicity of drugs and nano-systems to L929 and HCT-116 cells. L929 mouse epithelial cells and HCT-116 cells were cultured in Gibco MEM medium and Gibco DMEM medium, respectively, supplemented with 10% fetal bovine serum and 100 μg/mL streptomycin in a humidified atmosphere with 5% CO_2_ at a constant temperature of 37 °C to the logarithmic phase. The cells were seeded in a 96-well plate at a density of 5 × 10^4^ cells per well. After 18 h of incubation, 20 μL of samples with different concentration gradients were added to each well. After 12 h of coincubation, the illumination group was irradiated with a near-infrared light for 10 min. After 12 h, 20 μL MTT solution (5 mg/mL) was added to each well in the dark, followed by 4 h of incubation. After incubation, the solution was carefully removed from the wells, and 150 μL DMSO was added to each well. After shaking in the dark for 10 min, the absorbance was measured using a microplate reader at an absorbance of 490 nm, and the ratio of the absorbance of the drug-co-cultured cells to the absorbance of the medium reference was calculated to measure the survival rate of various cells.

#### 4.5.9. Uptake of Nanomaterials by Tumor Cells

The uptake of nanomaterials by tumor cells and their targeting to HA were evaluated. HCT-116 tumor cells were used to assess cellular uptake (the cell surface contains CD44 receptors), and the cellular uptake of the HA-modified nanocarrier was compared to that of the non-HA-modified nanocarrier. HCT-116 cells were seeded into 6-well plates (containing 1 mL of culture medium) at a density of 1 × 10^6^ cells per well and grown for 24 h. Subsequently, 1 mL of PBS, DOX, MPDA-Fe(III)-DOX, and MPDA-Fe(III)-DOX-HA were added (the equivalent DOX concentration in each group was 10 μg/mL), followed by an incubation of 4 or 8 h. After adding the samples, the irradiated group was incubated with near-infrared light (808 nm, 2 W/cm^2^) for 10 min. After incubation, cells were washed three times with PBS and stained with 1 mL of Hoechst 33,258 for 25 min. After discarding the staining agent, the cells were again washed three times with PBS before being observed under a fluorescence inverted microscope.

#### 4.5.10. Statistical Analysis

The results were expressed as mean ± SD. Statistical analysis was performed using one-way analysis of variance (ANOVA) and *t*-test. *p* < 0.05 was considered statistically significant.

## 5. Conclusions

In this study, mesoporous polydopamine nanoparticles (MPDA) were prepared using the template method, and an integrated photothermal–chemotherapy platform (MPDA-Fe(III)-DOX-HA) was subsequently established. MPDA-Fe(III)-DOX-HA had a uniform particle size of (133 ± 18 nm), a high drug-loading capacity for DOX (84.90 ± 0.68%), and a high release capacity at pH = 5.2 (release rate: 53.1%). Photothermal experiments revealed that the MPDA nanoparticles with surface-modified ferric ions had greater photothermal conversion ability and good photothermal stability. In addition, under local irradiation with an 808 nm near-infrared laser, MPDA-Fe(III)-DOX-HA exhibited strong cytotoxicity to HCT-116 cells, whereas targeted modification of hyaluronic acid reduced the cytotoxicity of nanoparticles to normal cells. The results showed that MPDA-Fe(III)-DOX-HA exhibited good biocompatibility and anti-tumor effect, which could be used as a reference for further research into photothermal–chemotherapy combination therapy in the future.

## Figures and Tables

**Table 1:** Drug loading capacity of nano-system with different DA to Fe(III) molar ratios.

Molar Ratios of DA:Fe(III)	6:1	3:1	2:1
Loading Capacity (LC)	80.41 ± 0.84%	84.90 ± 0.68%	81.87 ± 1.26%
Encapsulation Efficiency (EE)	16.08 ± 0.16%	16.98 ± 0.13%	16.35 ± 0.25%

## Data Availability

Not applicable.
